# Cremation in Europe

**Published:** 1883-03

**Authors:** 


### CREMATION IN EUROPE.

The gruesome story told the other day of a Russian peasant who was buried alive at Samara, and who was allowed to die in his grave because five hours elapsed before the necessary authorization could be procured for his disinterment, will increase the craving for cremation, which is more general than many people suppose. The thought of that five hours' death agony in the coffin, during which, says the record, "the poor fellow in his despair had bitten his fingers, torn his flesh and rent his clothing," will render more vivid the haunting horror which in some minds adds a new and indescribable terror to death. The peasant of Samara was buried when in an epileptic fit produced by drunkenness, and if he had been cremated death would have been instantaneous before consciousness could have returned. Those who entertain the dread of being buried alive will read with interest the able report on the memorial which the communal administration of Brussels presented to the Belgian Chamber, praying that cremation should be rendered opitional. The report of the committee on the petition, which was adopted with unanimity, and in whose prayer the Provincial Council of Brabant concurred, sets forth that at present cremation is not sanctioned by the Belgian law, although in the year 1798 or 1799 a father incinerated the body of his child in order to preserve its ashes in an urn. Italy, Germany, Switzerland and the United States have permitted cremation, and crematories have been established at Milan, Padua, Cremona, Lodi and Varese. At Milan, up to the end of 1881, 350 cremations had taken place, at a cost of £2 each. In 1799 Parisians were allowed the privilege of cremation on certain conditions, but the practice is now illegal in France, and a bill before the Chamber permitting every citizen the liberty of being cremated after death has not yet passed into law. The Belgian report enlarges on the great hygienic advantages of cremation, and maintains that it wounds neither the sentiment of human dignity nor the respect due to the mortal remains of our kind. It gives to death a conception, if not more consoling, at least more serene and more elevated, not only in ridding death of the associations of corruption and putrefaction, but also in symbolizing the transformation of being in the bosom of the purifying element, and the mysterious disengagement of the spiritual principle.—*Pall Mall Gazette.*

